# Interaction between the bone morphogenetic proteins and Ras/MAP-kinase signalling pathways in lung cancer

**DOI:** 10.1038/sj.bjc.6602790

**Published:** 2005-09-20

**Authors:** K S Kraunz, H H Nelson, M Liu, J K Wiencke, K T Kelsey

**Affiliations:** 1 Department of Genetics and Complex Diseases, Harvard School of Public Health, 665 Huntington Avenue, Boston, MA 02115, USA; 2 Department of Environmental Health, Harvard School of Public Health, 665 Huntington Avenue, Boston, MA 02115, USA; 3 Department of Neurological Surgery, Laboratory for Molecular Epidemiology, University of California at San Francisco, San Francisco, CA 94143-0441, USA

**Keywords:** bone morphogenetic proteins, methylation, epigenetic inactivation, *k-ras* mutation, non-small-cell lung cancer

## Abstract

Bone morphogenetic proteins (BMPs) are an integral component of the TGF*β* superfamily, responsible for regulation of cell proliferation, differentiation, migration and programmed cell death in a variety of cell types. The BMPs transduce their signals directly through the SMAD family of proteins but they also have been reported to interact with the MAPK and Erk pathways. Inactivation of the BMP pathway genes has been implicated as important in several cancers. Recent work has shown that *BMP3b* is epigenetically inactivated in cancer and suggests that *BMP6* can be epigenetically inactivated. We investigated whether *BMP6* is epigenetically inactivated in cell lines and whether *BMP3b* and *BMP6* are epigenetically inactivated in non-small-cell lung cancer (NSCLC). We also studied the relationship between BMP methylation and *k-ras* mutation. Here, we demonstrate that the *BMP3b* and *BMP6* genes are common targets of epigenetic inactivation in NSCLC, and that they are significantly more likely to be concurrently inactivated (*P*=0.009). Furthermore, this coinactivation of *BMP3b* and *BMP6* is significantly associated with mutation of *k-ras* codon 12 in lung cancer (*P*=0.003); those with a *k-ras* mutation were six times more likely to have concurrent methylation of these BMP loci. Hence, these data suggest that concurrent inactivation of the BMP and activation of the Ras signalling pathways are important in lung carcinogenesis.

*k-ras* mutation, which leads to aberrant Ras/MAP-K pro-growth signalling, has been well studied in cancer (Cunningham and Weinberg, 1985; Nelson *et al*, 1999; Huber and Stratakis, 2004). However, additional signalling pathways are acknowledged to play important roles in malignant disease, with the bone morphogenetic protein (BMP) signalling pathway emerging as important in multiple cancers (Nagatake *et al*, 1996; Tamada *et al*, 2001; Baldus *et al*, 2004; Dai *et al*, 2004; Kim *et al*, 2004). It is well recognised that the BMP signalling pathways are crucial for all stages of embryonic development, including regulation of lung development and airway branching (Weaver *et al*, 1999; Lu *et al*, 2001; Rosendahl *et al*, 2002). The BMPs have been shown to be growth inhibitory by suppressing proliferation (Dai *et al*, 2004; Haudenschild *et al*, 2004). They act through upregulation of the cell cycle proteins p21 and p27 (Nakamura *et al*, 2003; Brubaker *et al*, 2004; Haudenschild *et al*, 2004). The relationship between the TGF*β*/BMP antigrowth and Ras/MAP-K progrowth signalling pathways remains unclear, but there is recent evidence of crosstalk between them (Kretzschmar *et al*, 1997; Yue *et al*, 1999; Wach *et al*, 2001). While BMP-2 stimulation of osteoblasts leads to activation of Ras (Nakayama *et al*, 2003), in *Xenopus*, BMP-4-stimulated erythroid differentiation is inhibited by dominant-negative activation of the Ras pathway (Xu *et al*, 1996). SMAD7, one of the inhibitory SMADs of the BMP/TGF*β* signalling pathway, has been shown to cooperate with oncogenic Ras to escape growth arrest (Liu *et al*, 2003). While there is experimental evidence of an association between the BMP signalling proteins and oncogenic Ras, the nature of any interaction of BMP cytokine inactivation and *k-ras* mutation has been relatively unexplored in human tumours. As a result of the known importance of BMP signalling in the respiratory tract and the *in vitro* evidence of crosstalk between signalling pathways, we hypothesised that concurrent BMP inactivation and oncogenic *k-ras* mutation would be important in non-small-cell lung cancer (NSCLC).

## MATERIALS AND METHODS

### Study population

Eligible cases consisted of all the newly diagnosed patients with resectable lung cancer who received treatment at the Massachusetts General Hospital Thoracic Surgery Service from November 1992 to December 1996 (Nelson *et al*, 1999). All patients involved provided written informed consent in accordance with the appropriate institutional review boards. Patients with recurrent disease or nonoperable tumours were excluded. Tumours were snap-frozen in liquid nitrogen and stored at −80°C until genomic preparation. There were 155 cases with fresh tumour DNA available to be tested for BMP3b and BMP6 methylation. Demographic and epidemiologic data, including all of the data on tobacco use and occupational asbestos exposure, were obtained from a self-administered questionnaire completed by patients and subsequently reviewed by a single reviewer during the hospitalisation for thoracic surgery. Asbestos exposure was defined as having any occupational asbestos exposure (yes/no), assessed as previously described (Nelson *et al*, 1999).

### Cell culture

Lymphoblastic WTK1 and HT cells, small-cell carcinoma Shp77 cells, and non-small-cell carcinoma H322M and A549 cells were maintained in RPMI-1640 medium supplemented with 10% fetal calf serum. Prostate carcinoma DU145 cells and lung fibroblast IMR90 cells were maintained in minimum essential media (Eagle's) supplemented with 10% fetal calf serum. WTK1 cells (2 × 10^5^) in 10 ml media were treated with 1 *μ*l 5-aza-deoxycytadine in PBS or PBS alone for 3 days and then harvested.

### Reverse transcription PCR

Total RNA was isolated using Qiagen RNeasy kit. cDNA was synthesised from the RNA using the SuperScript III kit from Invitrogen. Reverse transcription PCR (RT–PCR) amplification of the BMP6 cDNA was performed using primers designed with Primer3. The BMP6 primers used are as follows: 5′-ACA GCA TAA CAT GGG GCT TC-3′ (sense) and 5′-CTC GGG GTT CAT AAG GTG AA-3′. B-actin cDNA was amplified using primers supplied with the SuperScript III kit. Approximately 50 ng of DNA was used as template. The PCR mixture contained 10 × PCR buffer, dNTPs (200 *μ*M of each), primers (1 *μ*M of each) and 0.25 *μ*l TaqGold. cDNA was amplified using an annealing temperature of 60°C over 35 cycles. PCR product was visualised on an ethidium bromide-stained agarose gel.

### Methylation-specific PCR

Two sets of primers specific to the BMP6 promoter region were designed using Primer3 to amplify bisulphite-modified DNA; one specific for DNA methylated (M) at the promoter region and the other specific for unmethylated (U) DNA. The primers used are as follows: (M) 5′-GGT TTG TTG GGT AGT CGG G-3′ (sense) and GCC CCT CCC CAA ATC G-3′ (antisense) and (U) 5′-TTG GGT AGT TGG GTG ATT GTT-3′ (sense) and 5′-ACA CCC CTC CCC AAA TCA-3′ (antisense). Approximately 50 ng of sodium bisuphfite-modified DNA was used as template. The PCR mixture contained 10 × PCR buffer, dNTPs (200 *μ*M of each), primers (1 *μ*M of each) and 0.25 *μ*l TaqGold. Product was amplified using an annealing temperature of 60°C over 38 cycles. Nontreated and *Sss*I methylase-treated lymphocytes were used as controls. PCR products were separated by electrophoresis and visualised by ethidium bromide staining.

### Statistical analysis

SAS software was used for statistical analysis. Wilcoxon rank sum test and Fisher's exact test (or *χ*^2^ test) were used for continuous and categorical variables in univariate analysis, respectively. Multivariate logistic regression was conducted to estimate the relationship between methylation of BMPs, k-ras mutation and covariates that were statistically significant in univariate analysis and that were important biologically.

## RESULTS

### Methylation of *BMP6* in cell lines

**Figure 1:**
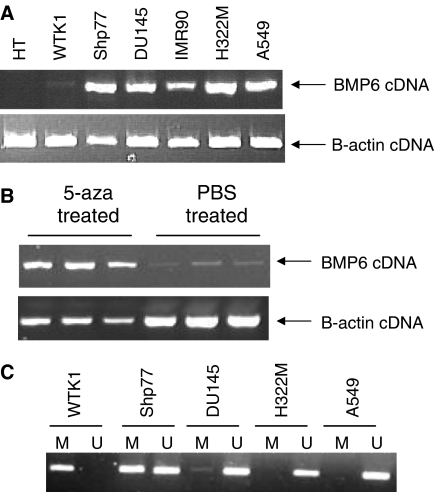
*BMP6* is epigenetically inactivated in cancer cell lines. (**A**) BMP6 cDNA was amplified using an annealing temperature of 60°C over 35 cycles with the following primers: 5′-ACA GCA TAA CAT GGG GCT TC-3′ (sense) and 5′-CTC GGG GTT CAT AAG GTG AA-3′ (antisense). The WTK1 and HT cell lines did not express BMP6. (**B**) WTK1 cells were treated with 1 *μ*l 5-aza-deoxycytadine in PBS or PBS alone for 3 days and then harvested. BMP6 is re-expressed in the WTK1 cells. (**C**) The *BMP6* gene promoter methylation status was determined by amplifying bisulphite-modified DNA using primers specific to methylated (M) and unmethylated (U) DNA. The DNA was amplified using an annealing temperature of 60°C over 38 cycles with the following primers: (M) 5′-GGT TTG TTG GGT AGT CGG G-3′ (sense) and GCC CCT CCC CAA ATC G-3′ (antisense) and (U) 5′-TTG GGT AGT TGG GTG ATT GTT-3′ (sense) and 5′-ACA CCC CTC CCC AAA TCA-3′ (antisense).

In an effort to determine whether transcription of the *BMP6* gene is inhibited by promoter methylation, we first examined the transcriptional status of *BMP6* in cell lines using RT–PCR (Figure 1A). BMP6 mRNA was absent in WTK1 normal human lymphoblastoid cells. Following treatment with 5-aza-deoxycytadine, a DNA methyltransferase inhibitor, transcription was reactivated, demonstrating that the loss of transcription of *BMP6* is due to epigenetic inactivation (Figure 1B). Using primers designed specifically to differentiate between methylated and nonmethylated DNA at the *BMP6* gene promoter, we demonstrated that the WTK1 cells are methylated at the *BMP6* gene promoter (Figure 1C).

### Methylation of *BMP3b* and *BMP6* in NSCLC cases

We evaluated *BMP3b* and *BMP6* gene promoter methylation status in 155 NSCLC cases. In a consecutive case series, we found that 57% (88 out of 155) and 43% (67 out of 155) of NSCLCs were methylated at the *BMP3b* and *BMP6* gene promoters, respectively. Since both BMP3b and BMP6 signal in the same pathways, we tested whether there was a relationship between inactivation of the two genes. We found that 52% (46/88) of the cases with *BMP3b* methylation were also positive for *BMP6* methylation; 31% (21/67) of cases without *BMP3b* methylation were positive for *BMP6* methylation. This statistically significant association between concurrent methylation of *BMP3b* and *BMP6* (*P*=0.009) indicates that the epigenetic silencing of these genes does not occur independently in lung cancer.

### Relationship between BMP methylation and *k-ras* mutation in NSCLC

We next investigated the relationship between BMP epigenetic inactivation and *k-ras* mutation. We previously have reported the *k-ras* mutation status of these cases (Nelson *et al*, 1999). In the subset of cases with both *k-ras* and BMP data, 16% (24/147) were mutated at codon 12 of the *k-ras* gene and *BMP3b* methylation was significantly associated with *k-ras* mutation (Table 1, *P*<0.03). Those with *BMP6* methylation were more likely to have *k-ras* mutation, but this was not statistically significant. However, concurrent methylation of both *BMP3b* and *BMP6* was strongly associated with *k-ras* mutation (Table 1, *P*<0.003).

### Concurrent BMP methylation, *k-ras* mutation, and tobacco and asbestos exposure in NSCLC

*k-ras* mutation has previously been associated with asbestos exposure in these patients (Nelson *et al*, 1999). In addition, pulmonary interstitial fibrosis (a condition associated with asbestos exposure) is associated with activation of the BMP/SMAD signalling pathway (Matsuse *et al*, 1996; Rosendahl *et al*, 2002). Therefore, we examined the relationship between tobacco use, asbestos exposure and *k-ras* mutation, and epigenetic silencing of the BMPs (Table 2). A logistic regression analysis, controlling for histology and gender showed that tumours with *k-ras* mutation are six-fold more likely to have concurrent *BMP3/6* silencing (OR=6.0, 95% confidence interval (CI) =2.0–17.9) and asbestos exposure significantly decreased the likelihood of concurrent *BMP3/6* methylation (OR=0.2, CI 0.1–0.9; Table 2). There was no statistically significant relationship between BMP methylation and tobacco exposure (Table 2).

## DISCUSSION

The BMPs are well recognised to play multiple crucial roles in the development of diverse tissues. They are also believed to play a role in the genesis of many tumour types, with their inactivation allowing for dysregulated cellular proliferation. There are multiple BMPs with overlapping substrate specificity (Stott *et al*, 1998). Our data, showing that epigenetic inactivation of *BMP3b* (at a frequency similar to that previously reported (Dai *et al*, 2004)) and *BMP6* tend to occur together in lung cancer, suggests that in NSCLC multiple BMPs may need to be silenced in order to abrogate their antigrowth signalling.

Increasing evidence shows that there is a crosstalk between TGF*β*/BMP antigrowth signalling and Ras/MAP-K progrowth signalling, and it is likely that the interaction of these pathways is cell type and tissue specific. Some evidence suggests that Ras inactivates SMAD signalling while other evidence suggests that Ras activates SMAD signalling (Kretzschmar *et al*, 1997; Yue *et al*, 1999). Further, inhibitory SMADs have been shown to be upregulated in coordination with *k-ras* mutation in epithelial cancer (Liu *et al*, 2003). Although the exact relationship between BMPs and Ras in signalling are unclear, our data are consistent with the mounting evidence that the relationship is important in solid tumours.

The finding that asbestos exposure is associated with a significantly different pattern of signalling dysregulation in NSCLC supports the notion that clonal selection has important nodal points that are influenced by the pattern of carcinogen exposure. The current work suggests that the Ras and BMP pathways are part of a complex network of inter-related and interacting signalling proteins where multiple factors, including carcinogen exposure, determine the precise character of somatic genes inactivated in this particular tumour, NSCLC. In the case of BMP inactivation, there was no apparent association of methylation silencing with tobacco exposure. However, we identified an inverse relationship between asbestos exposure and BMP methylation. Asbestos exposure has been associated with cytogenetic damage in humans (Dusinska *et al*, 2004). Hence, asbestos may be selecting for pathway inactivation via large-scale genetic changes. For example, SMAD4, the effector protein in the BMP pathway, has been identified as mutated and deleted in lung cancer (Nagatake *et al*, 1996; An *et al*, 2002). If asbestos were associated with induction of SMAD4 inactivation in lung cancer, one would expect the inverse association with BMP silencing that we have observed.

The balance between antigrowth and progrowth signalling that is imperative for normal cell development and homeostasis is reliant upon multiple feedback signalling pathways. *k-ras* mutation has been described as a relatively early event in cancer (Nelson *et al*, 1999) that constitutively activates the protein leading to active growth signalling. Our data show that the BMP pathway, which is antiproliferative, is selected for inactivation preferentially in *k-ras* mutant tumours in NSCLC, likely as a direct means of potentiating progrowth signalling. Direct experimental investigation of this interaction in human cells and a description of the effects of different carcinogens in different tissues upon the disruption of this signalling are clearly indicated. This will more precisely define the aetiologic effects of carcinogens (such as asbestos), delineate the interlocking pathways that are dysregulated in solid tumours, and potentially open avenues for prevention and treatment of these often-fatal cancers.

## Figures and Tables

**Table 1:** BMP methylation and *k-ras* mutation in NSCLC

	***BMP3B* methylation**			***BMP6* methylation**			**Concurrent *BMP3b* and *BMP6* methylation**		
	**NM^a^**	**M^a^**	***P*-value**	**NM^a^**	**M^a^**	***P*-value**	**NM^a^**	**M^a^**	***P*-value**
Overall prevalence in NSCLC (n (%))	68 (44%)	88 (56%)		96 (60%)	63 (40%)				
*k-ras mutation (codon 12)*									
Not mutated (%)	62 (91)	69 (78)		82 (87)	49 (78)		93 (89)	30 (70)	
Mutated (%)	6 (9)	19 (22)	0.03^b^	12 (13)	14 (22)	0.12^b^	11 (11)	13 (30)	0.003^b^

a NM=not methylated; M=methylated.

b χ^2^ test.

**Table 2:** Logistic regression modelling of BMP methylation and *k-ras* mutation in NSCLC

	***BMP3b* Meth**		***BMP6* Meth**		**Concurrent *BMP3b* and *BMP6* Meth**	
	**OR^a^**	**CI**	**OR^a^**	**CI**	**OR^a^**	**CI**
*k-ras* mutation	3.7	1.2, 11.4	2.9	1.1, 7.7	6.0	2.0, 17.9
Asbestos exposure	0.2	0.1, 0.6	0.3	0.1, 1.0	0.2	0.04, 0.9
Pack-years	1.002	0.992, 1.012	0.998	0.988, 1.008	1.003	0.922, 1.015

a Controlling for gender and histology.
